# A tree based approach for multi-class classification of surgical procedures using structured and unstructured data

**DOI:** 10.1186/s12911-021-01665-w

**Published:** 2021-11-23

**Authors:** Tannaz Khaleghi, Alper Murat, Suzan Arslanturk

**Affiliations:** 1 Department of Industrial and Systems Engineering, Wayne State University, Detroit, MI USA; 2 Department of Computer Science, Wayne State University, Detroit, MI USA

**Keywords:** Current procedure terminology (CPT) code, Machine learning, Ensemble learning, Importance weight, Random Forest, Multi-class classification, Surgery code

## Abstract

**Background:**

In surgical department, CPT code assignment has been a complicated manual human effort, that entails significant related knowledge and experience. While there are several studies using CPTs to make predictions in surgical services, literature on predicting CPTs in surgical and other services using text features is very sparse. This study improves the prediction of CPTs by the means of informative features and a novel re-prioritization algorithm.

**Methods:**

The input data used in this study is composed of both structured and unstructured data. The ground truth labels (CPTs) are obtained from medical coding databases using relative value units which indicates the major operational procedures in each surgery case. In the modeling process, we first utilize Random Forest multi-class classification model to predict the CPT codes. Second, we extract the key information such as label probabilities, feature importance measures, and medical term frequency. Then, the indicated factors are used in a novel algorithm to rearrange the alternative CPT codes in the list of potential candidates based on the calculated weights.

**Results:**

To evaluate the performance of both phases, prediction and complementary improvement, we report the accuracy scores of multi-class CPT prediction tasks for datasets of 5 key surgery case specialities. The Random Forest model performs the classification task with 74–76% when predicting the primary CPT (accuracy@1) versus the CPT set (accuracy@2) with respect to two filtering conditions on CPT codes. The complementary algorithm improves the results from initial step by 8% on average. Furthermore, the incorporated text features enhanced the quality of the output by 20–35%. The model outperforms the state-of-the-art neural network model with respect to accuracy, precision and recall.

**Conclusions:**

We have established a robust framework based on a decision tree predictive model. We predict the surgical codes more accurately and robust compared to the state-of-the-art deep neural structures which can help immensely in both surgery billing and scheduling purposes in such units.

## Background

CPT codes serve as the primary information component used to initiate several key processes in healthcare operations specially in surgical theatre. The CPT codes represent the detailed level of set of procedures in the surgery room. Knowing this code in advance of the surgeries enables the schedulers to make more accurate decisions while scheduling daily operations. In many disciplines, time component has been the primary input for operational planning [1–5]. Consequently, a major interest for the accurate prediction of the CPT codes is the ability to forecast the surgery durations. Given the CPT codes, the surgical service scheduler can plan for optimal number of surgical operations in a day with respect to resource constraints such as OR closing time [6], equipment availability, block specifications, and etc. Therefore, scheduling surgeries with accurate prediction of case durations [7] can considerably improve OR efficiency by decreasing patient wait times and surgical resource idle times.

Machine learning methods, as widely used tools for knowledge extraction from raw data, have been effectively used in supporting clinical decision making and safe delivery of increasingly personalized medicine. Similar to clinical decision support, various learning methods can also support human controlled predictions in the healthcare operations management such as procedural code prediction (CPT code in surgical units) for room assignment, duration forecasting, and scheduling. In surgical CPT prediction studies, surgery description holds invaluable information [8]. The text entry in the system of many hospitals is pattern-free surgery descriptions in surgical records. Therefore, any prediction attempt directly from unprocessed text results in high prediction error rates. In our prior study, we have identified, reduced, and structured feature sets by utilizing an unsupervised text mining approach from the free-text descriptions [5, 9].

The ongoing and standard CPT assignment approach involves manual labeling, which entails significant human effort and is cumbersome for huge surgery schedule databases in large hospitals. Current literature lacks methods that efficiently utilize the salient feature information embedded in the surgery descriptions in predicting surgery CPTs. While there are several studies using CPTs to make predictions in surgical services, literature on predicting CPTs in surgical and other services using text features is sparse. The only study utilizing text features for CPT prediction is performed by Jay [10]. Authors describe the construction of a neural network (NN) model for pathology CPT code prediction and its incorporation into their final verification step as a CPT code-checking application. They proposed a three-layer architecture for the NN model; a word-embedding layer, a bidirectional long short-term memory (LSTM) layer, and a densely connected layer. With the proposed NN-based prediction model, they predicted both validation and test set pathology CPT codes with accuracies of %97.5 and %97.6, respectively. Haq et al. [11] propose a state-of-the-art deep learning approach for predicting the surgery CPTs from the diagnosis codes (ICDs) entered by doctors. They outperform the rule-based probabilistic and association-rule mining based methods using a multi-label classification problem with distributed representation of inputs in high-dimensional sparse ICDs codes data. The trained model has a recall of 90@3 and a precision of 45@3. Accordingly, Li et al. build a log-regression and linear regression model to predict the surgeries based on the planned CPT codes [8]. Additionally, Levy et al. [12] represented 10 topics from diagnostic and procedural text information. The topics are aligned with pathologists, reports, and some CPT codes. XGboost and BERT models are used and compared with respect to CPT code, and signing pathologist prediction results. XGboost model outperforms the Bert model when predicting CPT code, however, models’ performances are similar when predicting signing pathologists. Surgery CPT prediction related studies include Lorenzi et al. where authors propose a predictive hierarchical clustering of CPT codes to improve prediction of a downstream regression model, i.e. surgical complications [13]. Their features include continuous lab values and binary indicators of patient history, such as whether a patient has diabetes. Another such study is the prediction of surgery durations using a log-regression and linear regression model based on the planned CPT codes [8].

Moreover, there’s also body of work on the automatic coding of diagnosis codes using textual and other information. For instance, Mullenbach et al. [14] propose an attention-based convolutional NN that predicts the codes solely from clinical text. Their method works by aggregating information across the text document using a convolutional NN, and selecting the most correlated clusters for each of the thousands of possible medical codes from an attention mechanism. Using this method, the achieved precision@8 of 0.71 and a Micro-F1 of 0.54. While there are similarities between our study and those predicting ICD codes using textual information, the pre-surgery textual data is significantly sparse. While majority of the recent studies in predicting ICD codes report on the success of neural network models over other classifiers, we herein use random forests due to their robustness, computationally simplicity, and ease at which they handle large numbers of descriptors.

In this paper, we develop an analytical predictive process to accurately identify the primary CPT codes for surgical operations. Surgeries intrinsically inherit a set of vital activities which can be characterized by a single or multiple codes that are maintained by the American Medical Association for uniqueness and consistency. Health centers employ globalized classifications of surgery types, in terms of so-called Current Procedure Terminologies (CPT). CPT codes are known as the most important factor in estimating the duration of the surgeries [15], as well as in preparing the surgical equipment, i.e., case carts. In the remainder of this section, we first give an overview of the developed approach. Next section, i.e., Methods section, details the CPT prediction models and their input data characteristics. Results section presents the performance of the proposed approach using a real-world data set. Discussion section highlights the salient aspects of the developed approach along with its limitations followed by our concluding remarks.

The historical surgical data set may contain multiple CPT codes for a completed surgery which collectively best describe the details of the procedures performed. Often these multiple CPT codes contain a primary and multiple secondary (i.e. add-on) CPT codes. Most data sets do not explicitly identify which CPT codes are primary as there may be multiple procedures completed. In this study, our aim is to identify only the most significant and dominant code for the planning of the pre-surgery or post-surgery services. In general, the primary CPT is the highest cost item that will be billed to the insurance or patient among other CPTs in the set, hence it is paramount that the reported CPT is accurately identified during the post-operation coding. In terms of scheduling, the surgery duration of the main CPT code of the procedure is used in allocating the block time availabilities.

While this study benefits from using the extracted features from free-text in our previous research [9], it integrates them with other continuous and categorical features to classify the surgeries by the primary CPT codes. Incorporating textual information available pre-surgery is shown to drastically improve CPT prediction [9]. In this study, given the multi-attribute feature space, the CPT codes are predicted by Random Forest (RF) classification model tuned with optimal parameters. The optimal set of parameters is obtained through an exhaustive search over user-specified parameter values for a set of estimators that significantly play role in enhancing the prediction output. The RF model, as a probabilistic classifier, assigns prediction probabilities to each class (i.e., CPT). Rather than using the highest probability CPT prediction, in this study, we consider multiple candidates for the primary CPT prediction based on the highest probabilities.

Using the candidate CPT list obtained from RF, our methodology then uses a novel approach to recalculate the class weights and re-prioritize the CPTs based on these weights. The weighting schema heavily depends on the extracted text features from the noisy surgery descriptions and physician notes available pre-surgery. The metrics used in the weight calculation includes the RF probabilities, and text-related information such as frequency, similarity, and relative feature importance measure of each CPT code in the set. This novel re-prioritization, a salient aspect of the proposed methodology, serves as extension model for Random Forest to improve CPT classification accuracy where the prediction task depends highly on the textual information. By incorporating this step as a secondary learner post RF classification, we are able to generate more reliable primary CPT class assignment using the information available pre-surgery.

Due to population health characteristics and hospital’s surgical service attributes, the real-world datasets are often unbalanced in terms of CPTs causing predictive performance degradation for rarely observed CPTs. Further, some CPT codes have greater heterogeneity of patients and surgical features which leads the prediction with little to no signal [13]. For instance, the CPT code “93320” for echo-cardiography procedures possibly correlate with many surgery records and procedural-related characteristics which makes it hard for a learner model to correctly label. In comparison, the replacement of a battery or pulse generator procedure tends to be operated on a more specific group of patients, with nearly similar procedure characteristics so the model can better learn the relationships between the features and a CPT label. In our assessment of the proposed approach, we report the results using different filtering methods to better interpret the labeling model and demonstrate the power of using machine learning model in conjunction with a knowledge-based model featured information from noisy text data.

## Methods

### Input data

The input data for the proposed approach is composed of both structured and unstructured data available prior to the surgery day from the electronic health records and other information systems. Unstructured data, such as textual features of procedure description and notes, provide additional information while structured data alone is not sufficient. We extract the TFIDF scores of the terms (i.e., text features) by deploying our text mining and text feature extraction method [9]. The consistency of the data reflects how close the prediction will be to the ground truth labels obtained from post-surgery medical coding done by medical coding professionals. We first determine the input features (both categorical and continuous variables) for predicting CPT code in this research and create the set of feature composed of surgeon ID, patient’s age, surgical case type, TFIDF of surgical terms, and surgery scheduled duration. The surgery scheduled duration is included in the input features to capture the system’s intrinsic knowledge about the case complexity. When the predicted CPT is used to improve scheduling through better duration estimations, the scheduled duration is used as scheduler’s input to the duration estimation but the final duration estimation (with distributional information) is available through the predicted CPT. In other words, the scheduled duration input is considered as the preliminary estimate which may be revised as per the predicted CPT.

The ground truth labels (CPTs) are obtained from medical coding databases. For a majority of surgeries, CPT codes are not provided as a singleton, rather as a set of CPTs. In other words, the surgeries are represented in terms of single or in many cases multiple CPT codes, i.e., “CPT list”. This is because most surgeries have multiple components, some of which are standard procedures such as anesthesia, etc. and others are multiple procedures being done concurrently. This study’s methods aim at predicting singleton CPT codes for each surgery. The single CPT label reflects the most dominant procedure among other CPTs. To train the models, we extract the dominant single CPT from the CPT set by reviewing their corresponding Relative Value Unit scores. Relative Value Units (RVUs) are used to quantify the physician services for reimbursement in US Medicare system. The CPT which owns the highest RVU score is chosen as the dominant CPT in the surgery CPT set, referred as the primary CPT [16].

### CPT prediction models

#### Random Forest multi-class prediction model

**Fig. 1:**
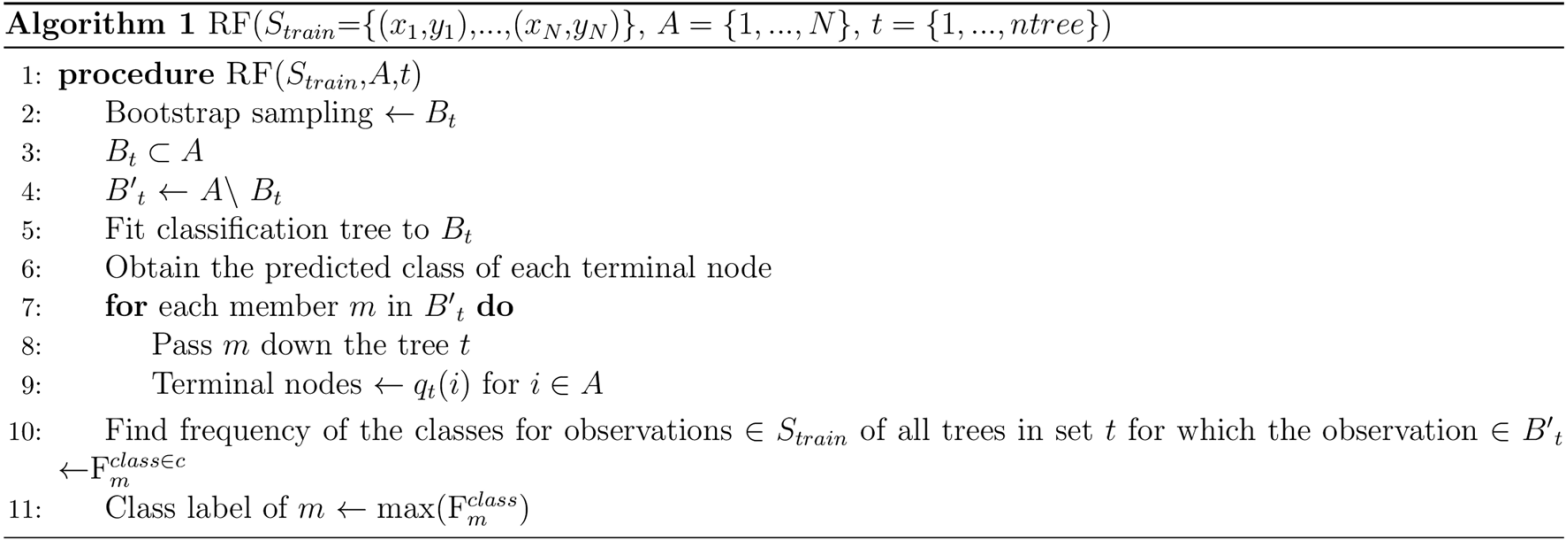
Random Forest classification algorithm

The first model predicting multi-class surgical CPT codes is the Random Forest (RF) [17]. This model is well-supported for the purpose of making decisions based on constructed probability tree of the possible scenarios and returning the class label which is the mode of the predictions given by the trees included in the ensemble. RF uses fully-grown trees in parallel as in an ensemble learning structure. The RF algorithm is represented in Fig. 1 [17].

**Fig. 2:**
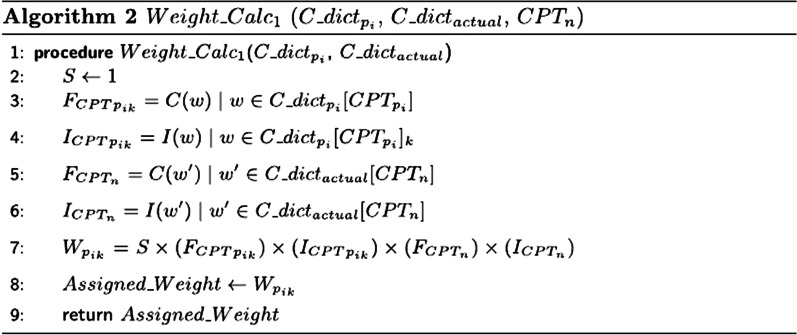
Algorithm for calculating class weight when word *w* is in both dictionaries, $CPT\_catalog\_dict_{p_i}$ and $CPT\_catalog\_dict_{actual}$ (named as $C\_dict_{actual}$ and $C\_dict_{p_i}$). *C*(*w*) and *I*(*w*) refer to the count and importance measure of the word *w*

**Fig. 3:**
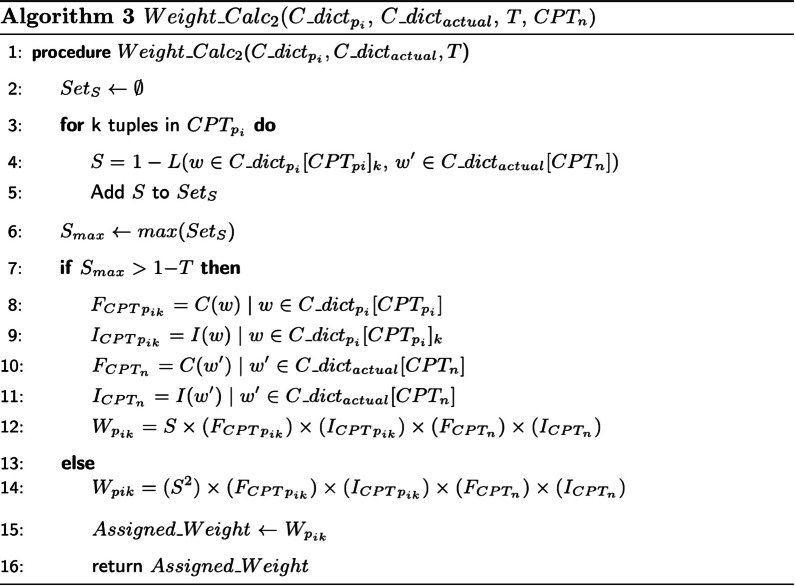
Algorithm for calculating class weight when word *w* exists in $CPT\_catalog\_dict_{p_i}$ but not in $CPT\_catalog\_dict_{actual}$ (named as $C\_dict_{p_i}$ and $C\_dict_{actual}$). *C*(*w*) and *I*(*w*) refer to the count and importance measure of the word *w*

In RF algorithm, the bootstrap samples are taken *N* times randomly with replacement at tree *t*. Moreover, in line 5 the classification process is acquired by splitting tree nodes of tree *t* on predictor variables, converging when reaching terminal nodes of the same class. In line 4, the class assignment of $B_t$ members in each node of tree *t* is obtained. The optimal class for observations in test set is the label with maximum frequency of the same training observations at terminal nodes of all constructed trees. The standard method for probability estimation is based on the proportion of trees that predict class *c* when *m* is passed down the tree *t* (See lines 8–12 of Algorithm 1).

#### Class weight recalculation method

We developed this method to apply it to the RF output to reduce the noise caused by those data points with small signal and thus improving the accuracy of CPT predictions. As an instance, CPTs of similar procedures are different codes but their textual feature contents are similar with minor differences. These differences are subtle and not resolved well in the ensemble model mechanism of the standard RF approach, thus require a special strategy to differentiate such observations. One solution is to design a CPT selection approach as a wrapper method which assigns weights to the most probable CPT sets. These CPT sets are in consistency with the most influential features, such as special characteristics of textual feature, feature importance measurements, and probabilities of the most likely CPTs. To build the wrapper, we need a model that informs the importance of text features and CPT assignment probabilities with respect to the classification task at hand.

In this approach, we used the RF’s feature importance measure as one of the factors playing significant role in weighting schema. RF calculates and generates this measure for the features based on the Gini impurity at each split node [18]. In the model, a feature’s importance is only defined if the decision tree model is selected as a base learner. However, such measures are usually accompanied by their pitfalls associated with data interpretation efforts. By incorporating the correlated attributes in the feature set, those features previously identified as significant become less important based on their assigned scores for some sparse CPT labels.

Accordingly, such importance measurements can be biased towards variables with more categories in the dataset. Instead of using these importance weights in coding the input text features and retraining the model, we make perturbations of the probabilities of the predicted CPT alternatives. Therefore, subsequent to the single CPT predicting task, we develop a novel perturbation-based approach to improve the accuracy of prediction using the class probabilities extracted from RF probability matrix ($p_{c}(x)$ of all *c* classes given each surgery case *x*). The prediction probabilities of the alternative CPT classes ($c\in C$) is then recalculated through a weighting scheme. In doing so, the probabilities of the CPT prediction alternatives are altered. This results in the modified ordering of the label predictions with respect to the importance of the CPTs in the class sequences based on calculated weights. Without loss of generality, we consider the top three predicted CPTs in this ordering. Given the ordered list of three CPTs with highest probabilities per surgery case, we represent $CPT_{p_i}$ where $i=\{1,2,3\}$ and $i=1$ denotes highest probability CPT and $i=3$ denotes lowest probability CPT, then we have:

1 $$\begin{aligned} CPT_{p_{i}{n}}=\left[ CPT_{p_1},CPT_{p_2}, CPT_{p_3}\right] _{n\in \{1,...,N\}} \end{aligned}$$

Let $CPT\_catalog\_dict_{p_i}$ be the dictionary of tuples, $\left[ (W_1, F_1, I_1),...,(W_k, F_k, I_k)\right]$ for *k* words in $CPT_{p_i}$ description word list, where *W*, *F*, *and I* represent word in CPT description, frequency of W, and importance measure of W, respectively. Then we have:

2 $$\begin{aligned}&CPT\_catalog\_dict_{p_i} = \{CPT_{p_i}: \{(W_1, F_1, I_1),..., \nonumber \\&\quad (W_k, F_k, I_k)\}_{k\in CPT_{p_i}} \}\forall i\in \{1,2,3\} \end{aligned}$$

In addition, let $CPT\_catalog\_dict_{actual}$ be the dictionary of tuples, $\left[ (W_{n1}, F_{n1}, I_{n1}),...,(W_{ne}, F_{ne}, I_{ne})\right] _{n\in \{1,...,N\}}$ for *e* words in $CPT_n$ description word list. Then we have:

3 $$\begin{aligned}&CPT\_catalog\_dict_{actual} = \{CPT_{n}: \{(W_1, F_1, I_1),..., \nonumber \\&\quad (W_e, F_e, I_e)\}_{e\in CPT_{n}} \forall n\in \{1,...,N\}\}\} \end{aligned}$$

The weighting approach supports the improvement of CPT prediction accuracy by incorporating pairwise similarity (*S*), term frequency (*F*), and term importance measure (*I*) (subset of RF feature importance matrix). Levenshtein distance between words *w* and $w'$ ($L(w,w')$) is calculated to further compute the pairwise similaries in Algorithm 3. Algorithms 2 and 3 calculate the class weights based on given variables. The coexistence of words in $CPT\_catalog\_dict_{actual}$ and $CPT\_catalog\_dict_{p_i}$ determines which algorithm should be used to compute the new weights for the most probable CPT assignments. Note that $CPT\_catalog\_dict$ has been addressed in Figs. 2 and 3 (Algorithms 2 and 3) as $C\_dict$. The ultimate weight is calculated through the following relational formula:

4 $$\begin{aligned} W=function(S_{w,w'},F_w,I_w,F_{w'},I_{w'}) \end{aligned}$$

The magnitude of the calculated weights increases if the importance and frequency measures also increase, $W \propto I \times F$. The reason is that if the frequency of a word feature in the learning model is boosted, gini impurity of this feature increases too. This is because it provides more classes in gini computation compared to the case when a less frequent feature is present. Additionally, pairwise word similarity measure can significantly improve the weights as the co-occurrence of the medical terms in the candidate CPT and actual label descriptions reflects the level of similarity in both CPT procedures.

Figure 2 represents the iterative procedure for each high-priority CPT in $CPT\_catalog\_dict_{p_i}$ ($i=\{1,2,3\}$) and each observation in $CPT\_catalog\_dict_{actual}$ ($\hbox {n}=\{1,...,\hbox {N}\}$) until the new weights for three high-priority CPTs of each surgery case is calculated. With N observations in surgery schedule, we can construct $I \times N$ matrix consisting of 3 weights for each observation. Lines 3 and 5 are computing the frequency count of the words *w* and $w'$ in $CPT\_catalog\_dict_{p_i}$ and $CPT\_catalog\_dict_{actual}$ dictionaries (refered as $C\_dict_{actual}$ and $C\_dict_{p_i}$ in the algorithm), namely $F_{{CPT}_{p_{ik}}}$ and $F_{CPT_n}$, respectively (Note that $w=w'$). Additionally, in lines 4 and 6 the importance measure of these words are extracted from importance matrix in RF model.

Figure 3 reflects the same behaviour with this difference: $w \ne w'$. In the light of this contrast, we can claim that $I_{{CPT}_{p_{ik}}} \ne I_{CPT_n}$. The Levenshtein distance approach [19, 20], is used to find the word pairs with maximum similarity measure and bring them as a key ingredient into the weight calculation. This distance method calculation is represented as $L(w,w')$ in Algorithm 3. The *T* parameter is a threshold distance measure defined specifically for each specialty based on the level of dissimilarity in description words [9].

**Fig. 4:**
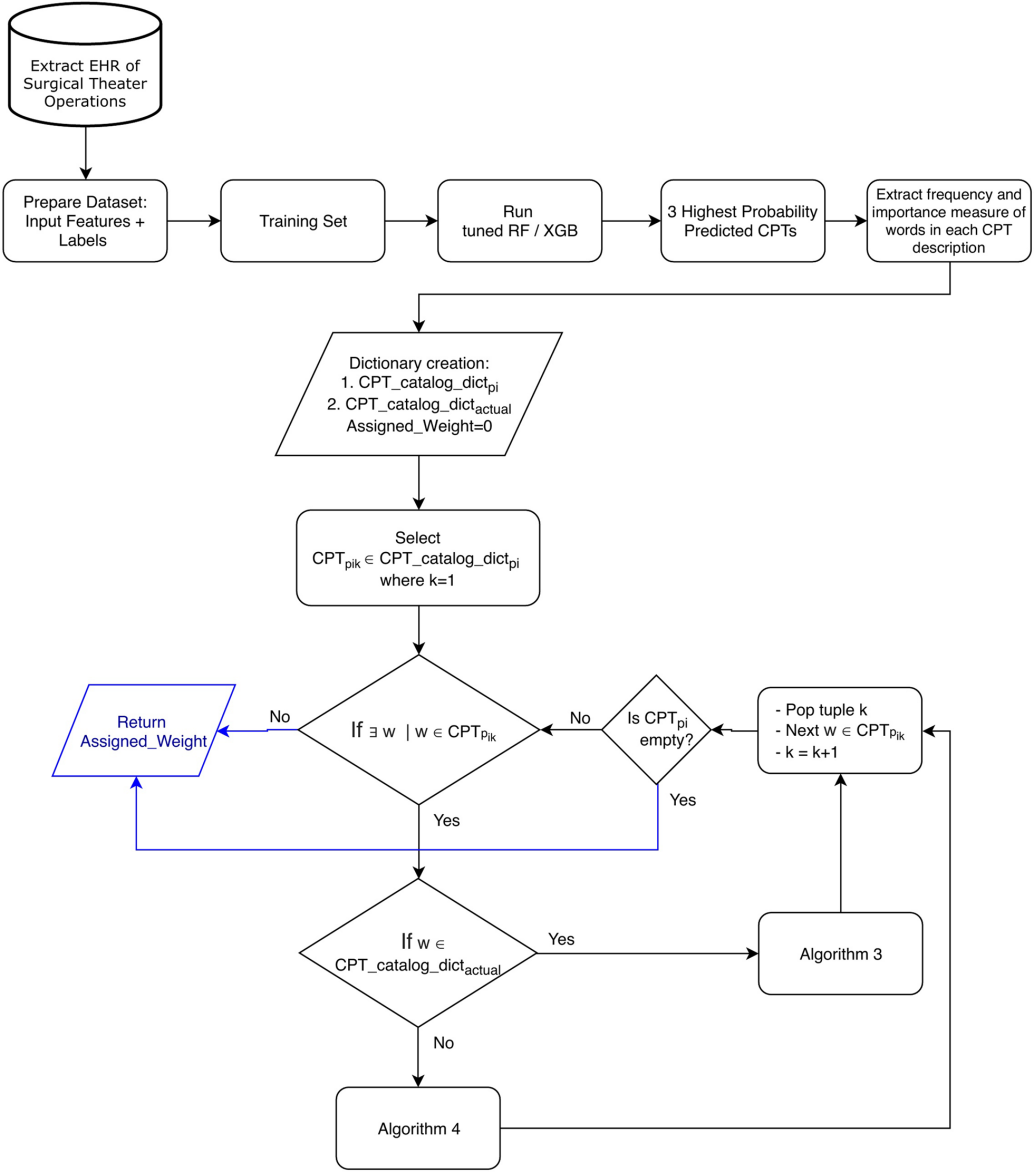
The class weight recalculation procedure. Algorithms 2 and 3 are calculating new weights based on the observed CPT instances

We illustrate the framework of our weight assignment approach in Fig. 4. The feature set is reprocessed and the CPT labels are assigned to each case using RVU measures. We take 80% of the entire dataset of each specialty for training the classification model and obtaining two dictionaries: $CPT\_catalog\_dict_{p_i}$ and $CPT\_catalog\_dict_{actual}$. The model is tuned using grid search technique to find the optimal combination of hyper parameters. Given the CPT probability matrix, feature importance matrix (outputs of the fitted model), and training data, we extract importance measure and occurrence frequency of the words in the descriptions for three CPT assignments with highest probabilities, as well as the actual CPT label. The framework presented in Fig. 4 executes two algorithms given the word co-occurrence states. We repeat this process for each of the three CPT codes in $CPT\_catalog\_dict_{p_i}$ dictionary and obtain their new weights. Given the calculated weights, we then determine a new order of the high-priority CPT codes and offer a CPT assignment for each surgery case which improves over the single CPT prediction output.

**Fig. 5:**
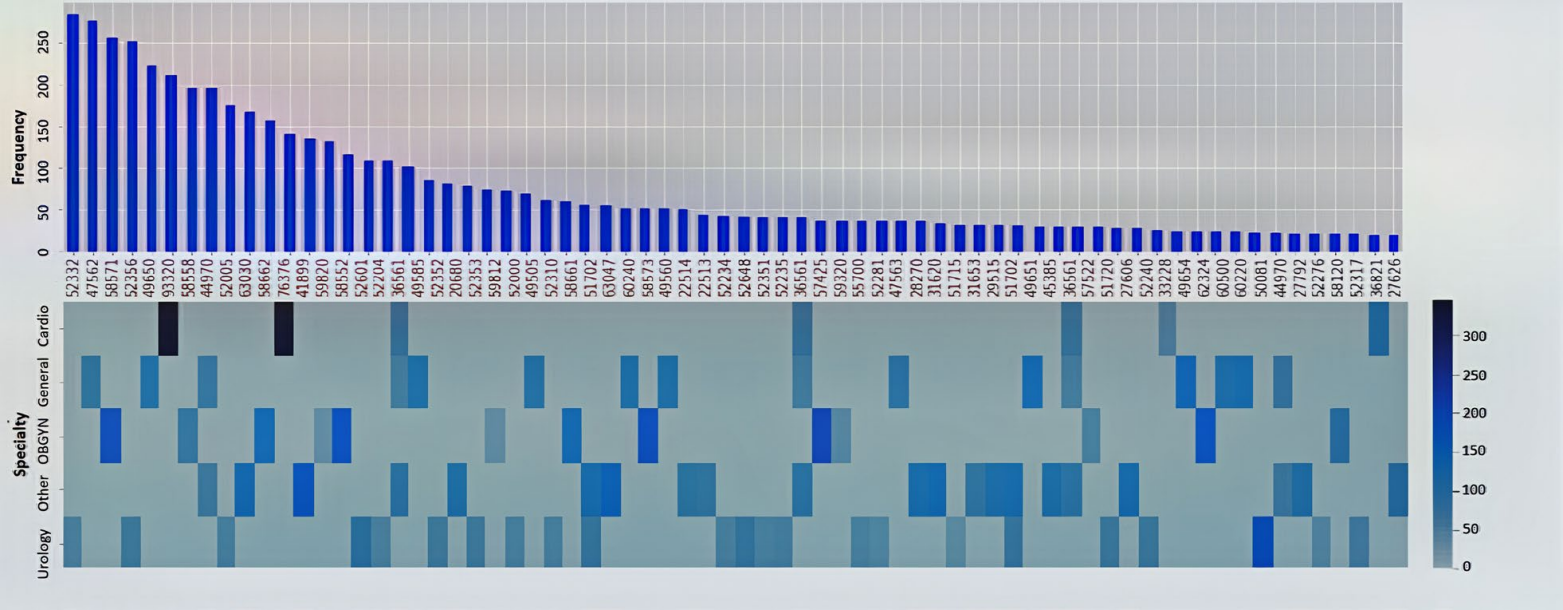
**a** The top figure shows CPT labels distribution for CPT codes with frequency greater than 20 in specialty data. **b** The bottom figure represents the average scheduled duration of CPTs with frequency more than 20 in each specialty dataset

**Fig. 6:**
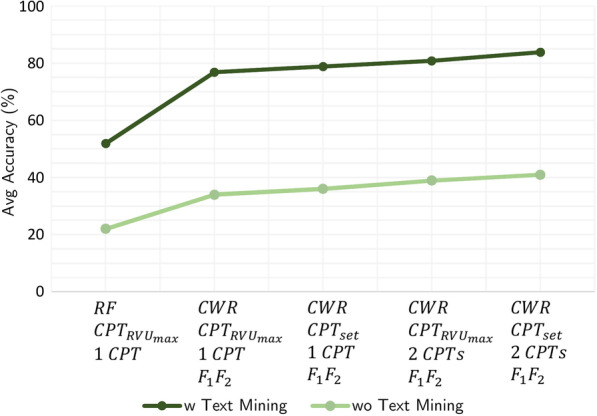
Plot of average CPT prediction accuracy under optimal settings

## Results

In our evaluation of the proposed approach, we used historical surgery datasets from a Southeast Michigan hospital’s surgery database. The data set contains about 28,000 of records of surgery scheduled cases, covering operations details in the period from May 2013 till June 2017 in the main OR suite. However, after performing initial data cleaning, the size of the data set is reduced to 10,000 cases. Then, the data set is split into five sections with regard to five specialty groups; Cardio, General, Urology, OBGYN, and other (specialties). We have 891 unique CPT codes in the full dataset. We have 891 unique CPT codes in the full dataset. Figure 5 shows the CPT codes with the frequency of greater than 20 in specialty datasets which illustrates the distribution of most significant CPT codes. The average scheduled durations of these CPT codes are also demonstrated in the heatmap plot for each specialty. As an example, in Cardio specialty CPTs “76376” and “93320” are labels with highest average scheduled durations.

The proposed multi-step framework consists of predicting the CPT codes using multi-class RF model and improving the accuracy using a novel re-weighting approach; called Class Weight Recalculation (CWR) method. For the performance evaluation of both phases, prediction and improvement are evaluated by the accuracy score. The predicted class is labeled as surgery CPT code given the specified input set. Initially, multi-class RF method has been fitted on each specialty data.The specialty “Other” can be decomposed into specialty types (e.g. ENT, Orthopedics, and etc) as more future observations of the same discipline are added to the data set. The CWR method reorders the high probability CPTs based on their new weights and revise the predicted CPT where applicable. The accuracy measures of both steps is reported in Table 2. We also compared the accuracy of these steps in our analysis with a baseline model; LSTM model from [10]. As the CWR method, complementary to the RF, improved the CPT prediction accuracy on average 8% across all specialties and proposed filtering and comparison schemas. This method also outperforms the NN model [10].

The various filters used in reporting the performance measure are explained in Table 1. These filters are utilized to better represent the nature of data and prediction schemes with respect to the proposed methods in multi-step CPT prediction pipeline. The filtering conditions, $F_1$, and $F_2$, enable us to investigate the capability of the proposed approaches in labeling the surgery cases with the most accurate CPT at different levels of interpretability. More specifically, $F_1$ allocates the medical code’s neighbors (i.e., CPT code differences less than 10) to the same group of procedure. The rationale is that the CPT codes with differences less than the threshold in $F_1$ share similar major procedure details such as approximate surgery area in the patient’s body and major action items planned by the physician. However, the small procedure differences can still lead us to variant codes.

On the other hand, due to the ambiguity in identifying the primary CPT code as prediction labels using RVU measure, the accuracy is calculated by comparing the predicted CPT(s) to the primary CPT as labeled based on RVU ($CPT_{RVU_{max}}$) or to all CPTs in the CPT set ($CPT_{set}$. Also, the prediction task can produce either a single CPT or two CPTs as predicted label(s) given the sorted CPTs with respect to the new weights calculated using CWR. This enables us to evaluate model by calculating accuracy@1 and accuracy@2 measures. With a single CPT or two CPTs prediction, the accuracy is calculated based on whether any of the predicted CPTs (or the single predicted CPT) exist in the CPT set (or is equal to $CPT_{RVU_{max}}$). Given these variations in filtering and comparison approaches, the accuracies are computed and reported in Table 2. The *C* column (complete data) accuracy can be considered as the baseline performance for RF and CWR methods while the same measures are represented for the baseline NN model as well.

**Table 1:** Different data filtering methods for exploratory result analysis

Filter label	Description
C	Complete dataset
$F_1$	Overlook small CPT difference $\tiny {(CPT_i - CPT_j\le 10 \Rightarrow {y_{0,1}=1})}$
$F_2$	Eliminate rare CPT occurrences $\tiny {(F_{CPT_i}<4)}$
$F_1F_2$	$F_1$ & $F_2$ $\tiny {(F_1 \cap F_2)}$

**Table 2:** CPT prediction accuracy measures under different filter combinations and accuracy calculation approaches for each specialty

Algorithm:	Random Forest															
Compare to:	CPT_RVUmax								CPT_set							
Compare with predicted:	1 CPT				2 CPTs				1 CPT				2 CPTs			
Filters:	C	F1	F2	F1F2	C	F1	F2	F1F2	C	F1	F2	F1F2	C	F1	F2	F1F2
Cardio	45	53	57	62	49	59	58	71	50	54	60	67	54	62	60	72
General	68	78	77	87	71	73	76	81	69	78	79	88	71	73	76	81
Urology	52	62	56	71	55	67	63	72	54	63	56	71	57	69	65	74
OBGYN	57	74	64	81	59	74	65	82	58	74	67	82	61	74	68	84
Other	36	51	54	68	37	53	56	69	38	52	56	69	40	54	58	70
Average	52	64	62	74	54	65	64	75	54	64	64	75	57	66	65	76

Algorithm:	Complementary weight recalcultion															
Compare to:	CPT_RVUmax								CPT_set							
Compare with predicted:	1 CPT				2 CPTs				1 CPT				2 CPTs			
Filters:	C	F1	F2	F1F2	C	F1	F2	F1F2	C	F1	F2	F1F2	C	F1	F2	F1F2
	54	61	68	72	68	76	86	89	55	62	69	73	69	75	86	88
	70	79	79	88	74	81	84	90	71	79	80	88	76	83	84	91
	57	66	62	76	60	74	62	78	58	67	71	78	63	74	74	79
	63	77	70	83	68	80	76	84	66	79	72	85	75	83	82	90
	39	52	55	65	40	53	55	66	44	57	58	71	44	58	59	71
	57	67	67	77	62	73	73	81	59	69	70	79	65	75	77	84

Algorithm:	Neural Net Model															
Compare to:	CPT_RVUmax								CPT_set							
Compare with predicted:	1 CPT				2 CPTs				1 CPT				2 CPTs			
Filters:	C	F1	F2	F1F2	C	F1	F2	F1F2	C	F1	F2	F1F2	C	F1	F2	F1F2
	39	45	48	54	39	46	49	54	39	46	50	56	39	47	51	57
	15	28	16	30	15	28	17	30	16	29	18	31	16	30	19	33
	20	24	22	26	20	24	22	26	22	24	23	28	23	26	23	28
	34	47	37	50	34	48	38	51	34	47	39	53	36	47	39	55
	17	20	22	25	17	20	23	26	17	21	23	27	17	22	25	27
	25	33	29	37	25	33	30	37	26	33	31	39	26	34	31	40

To further explain the proposed CPT prediction model (CWR) performance, we calculate the weighted average precision and recall. Given that the full dataset is unbalanced and has more than 800 unique CPTs, these performance metrics are weighted accordingly in each specialty dataset. Then, we compute the overall precision and recall given the specialty-specific performance measures and weight them based on the data sizes. The overall weighted average precision and recall for the Neural Net model (as baseline model) are 0.23 and 0.22 and for CWR are 0.45 and 0.52, respectively. These measures are calculated while prediction results are drawn from complete data (C), and the model predicts the primary CPT (accuracy@2). The true label is also considered as maximum RVU CPT. Since F2 is one of the substantial filters in the represented results in Table 2, we incorporate the precision and recall of NN and CWR with respect to this filtering method. With F2 filter, the precision and recall of NN are 0.28 and 0.26 and for CWR are 0.62 and 0.64 which are close to their corresponding accuracy measures shown in Table 2. While the highest accuracy (84%) is reported as accuracy@2 when compared with CPT set and existence of F1 and F2 filtering techniques, the precision and recall are also computed as 0.85, and 0.86, respectively.

Moreover, the text mining approach improves the CPT prediction performance and hence the surgery durations significantly. The average accuracy measures of multi-step CPT prediction approach are calculated and shown in Fig. 6; once with text transformed features and another time without involvement of text features in feature set.In terms of computational effort, training the model represented in CPT prediction framework takes between 1 and 5 min for different specialties; quickest is with Cardio specialty dataset and longest is with Urology specialty dataset. Scoring process of all specialties is negligible with respect to the run-time.

## Discussion

Some surgical cases in the analysis data are labeled with multiple CPT codes which implies the exact procedures performed. Among multiple CPT codes one is identified as the primary CPT using the RVUs and provided to the prediction model. We develop a multi-step prediction approach to predict the primary CPT codes for surgery cases. Such codes are used significantly in financial department for billing purposes, as well as in surgery department for scheduling operating rooms, preparing surgical equipment, etc. The goal of this study is to predict the dominant code for the pre-surgery or post-surgery service planning. We bring in our valued extracted text features from free-text EHR in our previous research, and integrate them with other continuous and categorical features; surgeon identifier, case type, patient info, etc. Incorporating textual information available pre-surgery is shown to drastically improve CPT prediction. Then, as the initial step, the CPT codes are classified by Random Forest model which is tuned with optimal parameters. The optimal model parameters are obtained in an exhaustive search over predefined parameter sets for the model estimators that significantly enhance the prediction results. The probabilistic RF model, provides us with the probabilities of all CPT classes in the dataset. Instead of using the highest probability CPT prediction which is the most common method of picking a class, we consider more than a single candidate based on the highest probabilities and calculate class weights using a novel approach to re-prioritize the candidate CPTs. The second prediction step heavily relies on the extracted text features from the unstructured surgery text data such as procedure descriptions. The drivers of the novel weighting schema are CPT probabilities, word frequencies, text-wise similarity, and relative feature importance measures. This re-prioritization approach improves the model performance where the CPT prediction task weighs more on the information extracted from textual data. Due to the sparsity of some CPT labels in the data, we report the results using different filtering methods to better represent the performance improvement in applying a probabilistic machine learning model in conjunction with featured information from an advanced knowledge-based model that we developed from noisy text data.

The proposed multi-class multi-step prediction model’s loss function only considers the CPT class accuracy, i.e. it does not consider the effect on the subsequent duration determination. To address this gap, We need to change the loss function to also incorporate the duration estimation accuracy. This can improve the prediction task where the CPT model fails to predict the accurate CPT with respect to the scheduling purposes. Moreover, this model only predicts the primary CPT with representing 1 or 2 candidate CPTs in the results, while predicting all CPTs representing the set of all procedures in surgery should improve the precision of surgery duration estimation which serves well in fulfilling the scheduler needs.

## Conclusion

CPT prediction study in surgical unit serves both the patients and staffs well. The better the prediction results are, the more reliable the surgery schedule is in terms of reducing over- and under-utilization. By knowing the CPTs pre-surgery, the hospital can efficiently prepare the equipments and reduce the risk of delays in such units. We proposed a multi-step framework: first predicting procedure CPT codes across all specialties using RF model with tuned hyper-parameters and second, recalculating the CPT weights using significant measures and rearrange them based on the new importance weights. Evaluation shows that the complementary learner improves the RF method results on average by 8%. The proposed multi-step prediction model predicts the CPT labels (2 most probable CPTs) with an average accuracy of 84% with respect to relevant and applicable filtering techniques which shows promising improvements compared to similar efforts. Moreover, the text features incorporated in the feature set improved the accuracy of prediction by 20–35% implying that the unstructured noisy text data improved the model knowledge about learning CPTs and enabling it to predict them more accurately. In terms of future enhancement opportunities, each surgery can be represented by more than one CPT (primary CPT) which highlights the need to predict multiple CPT codes. The outcome further explains the essential details of surgery description as well as improving the prediction of surgery duration distributions. While primary CPT still holds major procedure information, additional CPTs provide supplemental information which distinguishes the surgery cases from other similar ones. Furthermore, the use of SHAP feature importance in class weight calculation procedure may improve the prediction outcome since RF feature importance scores are sometimes subject to inconsistencies specially when missing values are present in the data.

## Data Availability

The datasets analyzed during the current study are not publicly available due hospital data were used but simulated data are partially available from the corresponding author on reasonable request.

## Abbreviations

CPT: Current procedure terminology
CWR: Class weight recalculation
ENT: Ear, nose, and throat
EHR: Electronic health record
ICD: International classification of diseases
LSTM: Long short-term memory
NN: Neural network
OBGYN: Obstetrics and gynecologist
OR: Operating room
RF: Random Forest
RVU: Relevant value unit
TFIDF: Term frequency inverse document frequency
